# Anti-Tumor Activity of *Hypericum perforatum* L. and Hyperforin through Modulation of Inflammatory Signaling, ROS Generation and Proton Dynamics

**DOI:** 10.3390/antiox10010018

**Published:** 2020-12-28

**Authors:** Marta Menegazzi, Pellegrino Masiello, Michela Novelli

**Affiliations:** 1 Department of Neuroscience, Biomedicine and Movement Sciences, Biochemistry Section, School of Medicine, University of Verona, Strada Le Grazie 8, I-37134 Verona, Italy; 2 Department of Translational Research and New Technologies in Medicine and Surgery, School of Medicine, University of Pisa, Via Roma 55, I-56126 Pisa, Italy; pellegrino.masiello@med.unipi.it (P.M.); michela.novelli@med.unipi.it (M.N.)

**Keywords:** *Hypericum perforatum*, hyperforin, reactive oxygen species, pH regulation, tumor prevention, tumor therapy, apoptosis, cancerogenesis, inflammatory signaling, natural compounds

## Abstract

In this paper we review the mechanisms of the antitumor effects of *Hypericum perforatum* L. (St. John’s wort, SJW) and its main active component hyperforin (HPF). SJW extract is commonly employed as antidepressant due to its ability to inhibit monoamine neurotransmitters re-uptake. Moreover, further biological properties make this vegetal extract very suitable for both prevention and treatment of several diseases, including cancer. Regular use of SJW reduces colorectal cancer risk in humans and prevents genotoxic effects of carcinogens in animal models. In established cancer, SJW and HPF can still exert therapeutic effects by their ability to downregulate inflammatory mediators and inhibit pro-survival kinases, angiogenic factors and extracellular matrix proteases, thereby counteracting tumor growth and spread. Remarkably, the mechanisms of action of SJW and HPF include their ability to decrease ROS production and restore pH imbalance in tumor cells. The SJW component HPF, due to its high lipophilicity and mild acidity, accumulates in membranes and acts as a protonophore that hinders inner mitochondrial membrane hyperpolarization, inhibiting mitochondrial ROS generation and consequently tumor cell proliferation. At the plasma membrane level, HPF prevents cytosol alkalization and extracellular acidification by allowing protons to re-enter the cells. These effects can revert or at least attenuate cancer cell phenotype, contributing to hamper proliferation, neo-angiogenesis and metastatic dissemination. Furthermore, several studies report that in tumor cells SJW and HPF, mainly at high concentrations, induce the mitochondrial apoptosis pathway, likely by collapsing the mitochondrial membrane potential. Based on these mechanisms, we highlight the SJW/HPF remarkable potentiality in cancer prevention and treatment.

## 1. Introduction

Despite the enormous and enduring effort undertaken by biomedical scientists in the investigation of tumor pathogenesis and therapy, cancer remains the second leading cause of death worldwide (World Health Organization, 2019 https://www.who.int/health-topics/cancer#tab=tab_1). Among the numerous antitumor drugs approved in the last fifty years, a large percentage originates from natural products or their derivatives [1,2]. Actually, natural antitumor agents show a broad spectrum of mechanisms to inhibit cancer development, through reduction of proliferation rate of malignant cells, induction of apoptosis, blockade of invasiveness and neo-angiogenesis [2]. Furthermore, they generally display lower side effects than other antitumor drugs [3].

In this review, among various natural compounds endowed with antitumor properties, we want to aim attention at *Hypericum perforatum* L., also known as St. John’s wort, and its principal constituent hyperforin, by reviewing research advancements about their key molecular mechanisms and highlighting their remarkable potential in cancer prevention and treatment. 

## 2. Signaling Pathways in Cancer and Molecular Targets Susceptible to Therapeutic Intervention by Phytochemicals

### 2.1. Oncogenesis 

Oncogenesis involves dysregulation of proto-oncogenes or tumor suppressor genes, which upon mutation can modify key cellular processes linked to cell proliferation and its control [4]. Despite significant advances in cancer diagnostics, the detection of a tumor growth in an early stage remains difficult due to the not infrequent lack or paucity of symptoms for long time. Thus, prevention of the early events in carcinogenesis appears crucial for protection against tumor development. A primary preventive role can actually be played by the use of dietary phytochemical supplements which would help to shield genotoxic insults, reverse the promotion stage of multistep carcinogenesis, and also halt or retard the progression of transformed cells [5]. Phytochemicals can veritably prevent these processes by activating antioxidant and detoxification pathways and exerting an anti-inflammatory action [6,7].

There is nowadays a wide consensus that inflammation plays a relevant role in carcinogenetic events. Many experimental results document a pro-tumor activity of inflammatory mediators and epidemiological studies reveal that chronic inflammation predisposes to various types of cancer [8]. In the attempt to replace lost cells or repair damaged tissues, cytokines, chemokines, angiogenic factors and extracellular matrix-degrading enzymes might in some cases increase the risk of cell transformation and most often sustain high proliferation rate of transformed cells [8,9]. Among the factors involved in carcinogenesis-related inflammation, cytokines like tumor necrosis factor (TNF)-α, interleukin (IL)-1β, interferon (IFN)-γ, and IL-6, appear to play a pivotal role. Cytokine signaling results in the activation of transcription factors, in particular nuclear factor kappa-light-chain-enhancer of activated B cells (NF-κB) and signal transducer and activator of transcription (STAT)-1/3, which are points of convergence for several pathways promoting malignancy, as evidenced in many studies [10,11,12,13]. In particular, NF-κB is a coordinator of innate immunity and inflammation and has emerged as an important endogenous tumor promoter [14,15]. NF-κB also enhances expression of IL-6, that further elicits cell proliferation and inhibits apoptosis via STAT-3 signaling pathway [16,17]. 

Interestingly, a number of phytochemicals impair both inflammation and oncogenesis by inhibiting activation of transcription factors [6]. For instance, ginger extract protects against ethionine-induced rat hepato-carcinogenesis restraining TNF-α production and NF-κB activation [18]; astaxanthin exerts anti-inflammatory effects by hindering NF-κB signaling pathway and prevents hamster buccal pouch carcinogenesis [19]; ursolic acid and resveratrol inhibit skin tumor development by hampering pro-inflammatory cytokine expression and NF-κB/STAT-3 activation [20].

### 2.2. Tumor Growth, Angiogenesis, Invasiveness, and Metastasis

In the complex network regulating tumor development, the role of mitogen activated protein kinases (MAPK) activity appears crucial for tumor cell survival. Growth factors, such as epidermal growth factor (EGF), platelet derived growth factor (PDGF), and insulin growth factor, act through extracellular-regulated kinase (ERK)1/2 and protein kinase B (Akt) signaling pathways to foster cell proliferation [21]. Phosphatidylinositol-3 kinase (PI3K)/Akt/mammalian target of rapamycin (mTOR) pathway represents another critical signaling axis which supports tumor growth [22], also through stimulation of protein synthesis and angiogenesis [23,24]. These kinases have connections to each other and to downstream transcription factors [25]. For instance, kinase-induced NF-κB activation favors cell survival in most tumor types, also by enhancing the expression of anti-apoptotic genes [15,25,26]. Promotion of apoptosis in cancer cells is indeed considered a powerful and reliable therapeutic tool. Notably, in both cancer cell lines and experimental tumor models, several phytochemicals have been characterized as pro-apoptotic agents effectively curtailing tumor growth [27,28,29,30]. 

Furthermore, natural products counteract tumor progression by hindering malignant cells ability to invade and seed in other body sites [27,28,31]. Invasiveness and metastatic spread are complex phenomena involving interactions between malignant and inflammatory micro-environmental cells [32,33,34]. Actually, tumor-associated macrophages potently induce angiogenesis by producing plentiful vasoactive factors, such as IL-8, vascular endothelial growth factor (VEGF), basic fibroblast growth factor (bFGF), and PDGF [33,35]. Surrounding fibroblasts and macrophages share with tumor cells the ability to release proteolytic enzymes, like metalloproteinases (MMPs), elastase and trypsin, which degrade extracellular matrix and cause abnormal blood vessel permeability [36,37,38]. Moreover, proliferation and migration of lymphatic endothelial cells, leading to neogenesis or remodeling of lymphatic vessels, can favor cancer metastasis [39]. 

Scientific evidence suggests that the daily consumption of adequate amounts of bioactive phytochemicals improves cancer prognosis, as recently reviewed [40]. For instance, herbal extracts from *Astragalus membranaceus*, *Angelica gigas*, and *Trichosanthes kirilowii maximowicz* were reported to suppress lung metastasis in vivo through inhibition of IL-6/STAT-3 signaling pathway [40,41]. The ethanol extract of *Rhizoma amorphophalli* significantly decreased proliferation and migration of breast cancer cells in the lung [42]. Daucosterol linoleate downregulated the expression of VEGF, MMP-2 and MMP-9 in breast and lung cancers [43], as *Manuka* honey did in human colon cancer cell lines [44]. Thus, the treatment with various natural product extracts represents a promising approach to limit both progression and spread of tumors.

## 3. Reactive Oxygen Species as a Double-Edged Sword in the Fight against Cancer 

Free radical oxygen species (ROS) are highly reactive byproducts of cell metabolism. ROS level in normal cells is tightly regulated by redox homeostasis based on several antioxidant detoxifying agents [45]. However, if ROS amount exceeds a certain threshold, serious damages occur in cells, like DNA deletions, insertions, single- and double-strand breaks that, if not repaired, might lead to either tumor transformation or cell death [46].

**Figure 1:**
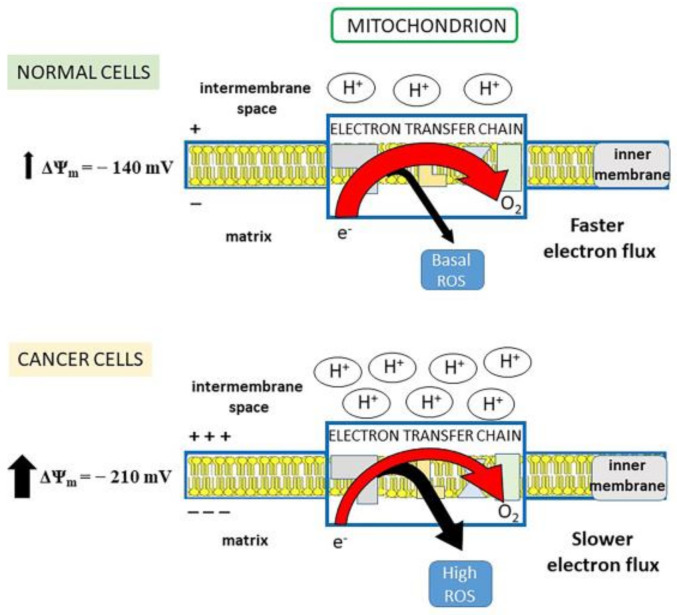
Electron flux in electron transport chain (ETC) and mitochondrial (mt)ROS production. Normal cells: the physiological electron flux in ETC determines robust proton pumping from the matrix to the intermembrane space and maintains stable negative mitochondrial membrane potential (Δψ_m_ = −140 mV). In this situation, electron transfer to O_2_ is fast and the electron leakage for ROS generation is small (basal ROS production). Cancer cells: the electron transfer to O_2_ is slow because the associated proton pump is impaired by the fact that protons must be pumped against a stronger electrochemical force (Δψ_m_ = −210 mV). Thus, a larger number of electrons leaks out ETC, increasing ROS production.

Mitochondria are considered the major production site of ROS, generated through the electron transport chain (ETC). The free energy made available by the electron transfer drives a proton-motive force (pmf) across the inner mitochondrial membrane. As protons are positively charged, pmf determines a charge gradient which constitutes the inner mitochondrial membrane potential (Δψ_m_). When the Δψ_m_ is high, like in cancer cells, the electron transfer is slowed down, as protons have to be pumped against an enhanced electrochemical force, so that an electron leakage and, consequently, high ROS production are favored [47] (Figure 1). By lowering the Δψ_m_ toward that of normal cells, the electron flow would accelerate with the result that (i) electrons spend less time in ETC, thus decreasing their chance of reducing oxygen to superoxide radical; (ii) the increase in oxygen consumption due to the faster electron transfer limits free oxygen availability for superoxide generation [47]. In this regard, tumor cells have a more negative mitochondrial membrane potential inside the matrix (Δψ_m_ ~ −210 mV), than normal cells (Δψ_m_ ~ −140 mV) [28,48] (Figure 1). 

Quickly after superoxide generation, other reactive species can be produced, including hydroxyl and thiol radicals, hydrogen peroxide (H_2_O_2_) and peroxy-nitrites, the latter derived from the interaction between superoxide and nitric oxide produced by inducible nitric oxide synthase (iNOS) [48,49]. Overexpression of iNOS is common in different tumor types [49,50] and in surrounding microenvironmental cells, due to cytokine-elicited activation of NF-κB and STAT-1 signaling [51,52].

Besides their direct production in mitochondria, superoxide radicals are enzymatically generated by NADPH oxidases (NOXs) located in plasmatic and organelle membranes [53], and can also arise as byproducts from COX-2 and 5-lipoxygenase (5-LO) activities. Anyway, superoxide radical can be easily converted into H_2_O_2_, that exhibits higher stability and target selectivity than all other reactive species [53,54]. Thus, H_2_O_2_ can operate as a bona fide second messenger for the intracellular transduction signaling of hormones, growth factors, cytokines and other inflammatory mediators, by oxidizing the thiol groups of cysteine residues of target proteins, including tyrosine phosphatases (PTPs) [49,54,55,56]. A generalized inhibition of PTPs can actually shift the dynamic equilibrium between PTPs and protein kinases, favoring kinase activation cascade [56]. 

A tight crosstalk between ROS, NF-κB and inflammatory mediators is documented. Pro-inflammatory cytokines, through binding to their receptors and the subsequent ROS/H_2_O_2_ signal transduction, drive the phosphorylation and activation of IκB kinases (IKKs) and downstream NF-κB [57,58,59]. NF-κB signaling is able to prevent ROS-elicited apoptosis by activating gene transduction for antioxidant enzymes, thereby further sustaining a pro-survival response [57,60]. Thus, despite enhanced ROS, malignant cells escape death by maintaining ROS levels below the lethal threshold through the production of large amounts of ROS scavengers. At the same time, cancer cells can utilize ROS signaling to enhance growth by promoting the activity of pro-survival kinases (i.e., PI3K, Akt, ERK1/2, mTOR) [48,61]. Additionally, high ROS levels can trigger angiogenesis through stabilization of hypoxia inducing factor (HIF)-1, that drives the expression of hypoxia-responsive genes leading to upregulation of MMP-1/2/9 and favoring tumor cell infiltration in other tissues [62].

There is increasing consensus that natural products, even at low concentrations, are able to decrease carcinogen-elicited DNA damage, thereby exerting tumor preventive effects through the regulation of ROS-related processes [45], although the mechanisms involved are still unclear. Low concentrations of natural agents are probably ineffective in vivo as ROS scavengers, because both normal and even more cancer cells possess an ample reserve of antioxidants, such as vitamin C and glutathione (GSH) [28]. It is more likely that natural products act at low concentration by interfering with specific steps of signaling pathways [28,63]. Other mechanisms may involve modulation of expression and/or activity of oxidative stress-related enzymes [64,65], as well as limitation of ROS generation through a partial dissipation of the inner mitochondrial membrane potential [28,45,66]. Conversely, it has also been suggested that, in the context of established cancer, several natural bioactive compounds, especially at high concentrations, can exert pro-oxidant rather than anti-oxidant activity, further enhancing oxidative stress and driving tumor cells to death [67,68]. This hypothesis originates from in vitro evidences that catechins and other natural products could actually induce ROS-dependent apoptosis in various types of tumor cells [69,70,71,72]. Anyhow, to explain the large variability of the experimental data reported in literature, a number of limitations of the in vitro studies on the anti-tumor effects of natural products should be mentioned: (i) the high concentrations of natural compounds often used in such studies raise the question whether they are really relevant to the in vivo situation [28]; (ii) the majority of the in vitro experiments has been performed with cell lines cultured in a medium often deficient in antioxidants, that can lead to overstate the beneficial effects of added antioxidant products [73]; (iii) some polyphenols are instable in standard culture media, in which they can generate oxidation products capable of depleting cellular glutathione and eventually resulting in pro-oxidant effects [73]. For these reasons, in vivo experiments are expected to make us better understand at the molecular level the real effects of natural products on the reactive oxygen radicals. Indeed, although it has been well documented that polyphenols and other natural compounds are able to inhibit tumorigenesis and tumor growth in several animal models, a putative ROS-dependent pro-oxidant mechanism in such in vivo inhibition has not been elucidated yet [67]. 

## 4. Dysregulation of Proton and Ion Content in Cancer Cells as a Possible Target for Phytochemical Therapy

Another hallmark of neoplastic cells is the aberrant regulation of ion flow affecting the intra- and extra-cellular pH homeostasis. Many channels able to modify proton concentration are present in the plasma membrane. The most important is the Na^+^/H^+^ exchanger (NHE) that uses the natural inward Na^+^ gradient to move Na^+^ into the cytosol and H^+^ in the extracellular space [74,75]. Remarkably, the expression of the ubiquitous NHE1 antiporter increases by two-three-fold in many different tumor types [76]. The key role of NHE1 in carcinogenesis has been well elucidated by Reshkin et al., who provided evidence that the earlier event in tumor development was the cytoplasmic alkalization consequent to the transformation-dependent activation of the NHE1 channel, driven by an increased affinity of NHE1 allosteric proton regulatory site [77]. In addition to protons, NHE1 activity can be amplified by osmotic or ischemic stress, as well as by growth factor and MAPK signaling [74,78,79].

Besides NHE1, pH can be regulated by the Na^+^/HCO_3_^−^ cotransporter, that is overexpressed in hypoxic tumors [74,80]. In addition, other plasma membrane channels are effectors of pH dynamics, such as the voltage-gated proton channel, the vacuolar type H^+^-ATPase (V-ATPase), the H^+^/K^+^-ATPase and the H^+^/monocarboxylate transporters, the latter extruding from the cell lactate or other monocarboxylate metabolites together with a proton [81,82]. Importantly, both pharmacological and phytochemical inhibitors of these channels were found to revert or at least attenuate the malignant phenotype of cancer cells, suggesting that a crucial role is played by pH and ion concentration changes in tumor development, as reviewed in [74,78,81,83,84].

Aside the pH effectors above described, a number of pH sensors (i.e., proteins whose activities or ligand binding affinities are regulated by pH) have been recognized [83]. Actually, changes in proton concentration (i.e., pH dynamics) are reflected by the amount of the protonated form of histidine residues which are able to buffer pH variations [84]. In this way, pH dysregulation can simultaneously modify charge and function of many protein targets, facilitating gain- or loss-of-function of mutated proteins as it occurs in the R337H substitution of the tumor suppressor p53 in cancer [84]. 

It should be reminded that in normal cells proton concentration is slightly higher in cytosol (pHi ~7.2) than in extracellular space (pHe ~7.4) [83]. Conversely, in tumor cells pHi increases to ~7.6 and pHe decreases to ~6.8, thereby reversing the pH gradient (ΔpH) direction. Such pH dysregulation, that occurs early in cancer development, is further exacerbated during tumor progression [83]. 

**Figure 2:**
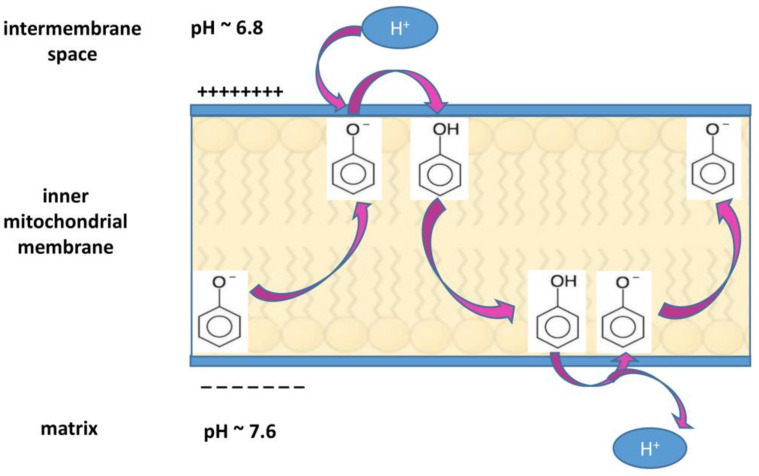
Mechanism of protonophore activity in the inner mitochondrial membrane. For its high lipophilicity, a protonophore like the phenolate anion depicted in the Figure (or hyperforin, as pointed out in the text) is going to be located inside the membrane and can move from one membrane-water interface to the other. As regards the inner mitochondrial membrane, a protonophore molecule captures a proton at the interface with the positive charged intermembrane space, where the proton concentration is high (pH 6.8). In the protonated form, it moves to the interface with the matrix, where the low proton concentration (pH 7.6) and the presence of negative charges favor proton extrusion, and it assumes an anionic form. This form does not interfere with its lipophilicity because it is stabilized by resonance, thus allowing its presence in the membrane and recycling. In this way, the protonophore can dissipate the transmembrane potential (Δψ_m_).

The alterations in proton concentration imply serious consequences in the cellular functions. The acidification of extracellular tumor microenvironment contributes to enhance invasiveness and metastatic spread, suppress immune responses and induce chemotherapy resistance, whereas the cytosolic alkalization favors cell proliferation, protects from apoptosis, and supports tumor neo-vascularization by promoting VEGF expression [74,81,83,84,85]. It has been observed that, if the dysregulation of proton concentration is reversed by inhibiting NHE1 transporter activity, the resulting intracellular acidification contributes to cell death via apoptosis [85,86,87]. Reciprocally, the observation that upon exposure to apoptotic stimuli, an intracellular acidification occurs in mammalian cells undergoing apoptosis [87] confirms the strong relationship between acidic pHi and apoptosis pathway [88]. As counteracting pHi increase and Na^+^ overload reverts cancer cell phenotype, it is worth to highlight that the use of proton and ion transporters inhibitors such as ameloride, cariporide and other synthetic inhibitors, can be a new promising therapeutic approach against tumor development [78,83]. In this regard, also some natural products can affect pH-regulating ions channels. For instance, ginsenoside induces apoptosis in hepatocellular carcinoma by decreasing NHE1 expression and activity, via EGF/ERK/HIF-1 signaling pathway [89]. Ellagic acid markedly down-regulates ROS formation and NHE1 expression leading to decreased NHE1 activity, pHi, glucose uptake and lactate release in endometrial cancer cells [90]. Corncob extract decreases proliferation and viability in glioma cells by reducing ROS amount, Bcl-2, and lactate monocarboxylate transporter 1 expression levels [91]. Moreover, the increase in plasmatic and mitochondrial membrane proton conductance could represent an alternative mechanism by which natural compounds can restore pH homeostasis without affecting a specific ion channel, but still allowing protons re-entry into the cytosol or the mitochondrial matrix. For instance, in the mitochondria, any compound acting to decrease differences in pH across the membrane, lowers Δψ_m_ and can also cause uncoupling of the electron flow with the oxidative phosphorylation [92]. In general, lipophilic, weakly acidic compounds can work as protonophores [92]. Due to their lipophilicity, they enter and remain in the membrane in which they can shuttle from one membrane-water interface to the other. For their weakly acidic nature, they can assume either the neutral state or the anionic form. In the latter form, stabilization occurs by delocalization of negative charge by resonance, so that their lipophilicity is not compromised. A protonophore in anionic form (e.g., a phenolate) is attracted by the positive side of the membrane-water interface close to the intermembrane space (where the pH is 6.8). There, it can bound a proton and move to the other membrane-water interface close to the matrix, where it releases the proton, due to the negative charge on this side of the membrane and the high pH (7.6) of the matrix. Then, it comes back for another cycle [28,92] (Figure 2). 

It is known that a slight increase of mitochondrial membrane proton conductance, without affecting ATP production (mild uncoupling), can inhibit ROS formation by reducing Δψ_m_ [93] (through the mechanism described in Figure 1). Such protonophore activity has been established for the acylphloroglucinol family of natural products, such as clusianone, isolated from the roots of *Clusia congestiflora*, which is a mitochondrial uncoupler and a well-known cytotoxic anti-cancer agent in hepatocarcinoma [94], the bis-geranylacylphloroglucinol moronone, contained in the *Moronobea coccinea* extract, that displays cytotoxic activity in breast cancer cells by dissipating the mitochondrial proton gradient [95], and hyperforin, from *Hypericum perforatum*, which is able to increase proton conductance of both plasma and organelle membranes [96], as it will be detailed below.

## 5. *Hypericum perforatum* or St. John’s Wort (SJW) and Hyperforin (HPF) 

SJW is an herbaceous plant containing many bioactive molecules [97]. For long time SJW has been used in the traditional medicine for its anti-inflammatory and lenitive properties and recently it has been mostly employed for the treatment of anxiety and depression to replace conventional antidepressant drugs [97,98], with which it shares the inhibition of the uptake of monoamine neurotransmitters as a mechanism of action [99]. Several randomized controlled clinical trials have been performed and their results indicate that SJW treatment of mild/moderate depression is effective and well-tolerated with low occurrence of adverse effects [98,100,101]. Besides such anti-depressant properties, other biological activities of SJW make this vegetal extract very suitable for both prevention and treatment of a number of diseases in which the inflammatory events play a relevant role. The pharmacological properties of *Hypericum perforatum* are due to a number of bioactive molecules, among which the most important and characteristic of this species are hyperforin (HPF) and hypericin, usually present in the total hydro-alcoholic SJW extract within a range of concentrations of 1–5% and 0.1–0.3%, respectively. Additional active compounds, such as hyperoside, rutin, quercetin, several catechins and other polyphenols are often contained in the total extract of SJW, although with a large variability of concentrations, mainly depending on seasonal fluctuations and the geographic origin of plant [102,103,104]. 

Among the major SJW components, we will not consider here the antitumor effect of hypericin, whose mechanism of action is based on its phototoxic properties, currently exploited in the so-called photodynamic therapy applied in several tumor types, especially of cutaneous origin [105,106]. We will actually focus on HPF, that is the primary active principle responsible for both the antidepressant and the anti-inflammatory properties of SJW [52,99,107,108,109]. 

HPF is a bio-active acylphloroglucinol abundantly present in the apical flowers of *Hypericum perforatum* plant. Notably, pharmacokinetic data, obtained in healthy volunteers, indicate that oral administration of anti-depressive therapeutic doses of SJW hydro-alcoholic extract (3 × 300 mg daily, containing 15 mg HPF) results in a maximal HPF circulating concentration of 0.28 μM, at 3.5 h after a single dose, and a steady-state concentration of 0.18 μM [110]. Similar results were obtained in a successive study [111], confirming the bioavailability of HPF and the achievement of suitable blood levels after ingestion of SJW extract. Altogether, the available data show that HPF contained in SJW extract is effectively absorbed and can protect cells against pro-inflammatory cytokine-induced damage. Moreover, it should be stressed that HPF is able to induce long-lasting changes in the cell signaling and transcriptional pathways even after being withdrawn from the cell culture medium following a pre-incubation period [112,113]. While HPF is stabilized by the presence of antioxidant compounds in the whole SJW extract, purified HPF is relatively unstable in presence of light and oxygen [114]. Therefore, various hyperforin salt preparations [110] and stabilized hyperforin analogs, such as DCHA-hyperforin [115,116] or aristoforin [117] were tested and proved to retain HPF biological activity.

### 5.1. Protective Effects of SJW and HPF against Noxious Stimuli

SJW extract and HPF exert powerful anti-inflammatory effects by blocking the activation of several signaling pathways triggered by injuring stimuli and by slowing down the production of inflammatory mediators. SJW and HPF have been shown to suppress the activities of COX-1 and 5-LO in vitro and in vivo [107,108]. Strong inhibition of prostaglandin E_2_ (PGE_2_) has been confirmed using either SJW extract in a lipopolysaccharides (LPS)-stimulated RAW 264.7 mouse macrophage model [118], or HPF in an in vitro assay of LPS-stimulated human whole blood [119]. In our laboratory, we observed that, in pancreatic β cells as well as in rat and human pancreatic islets, SJW and HPF are able to markedly inhibit both IFN-γ-elicited STAT-1 and TNF-α/IL-1β-triggered NF-κB activities, leading to prevention of iNOS gene expression and protection against β-cell damage [52,120,121]. Furthermore, in the same cells supplemented with SJW or HPF, we showed a significant concentration-dependent reduction of the phosphorylation level of several cytokines-elicited kinases, such as IKK, Akt, p38, c-Jun N-terminal kinase (JNK), ERK1/2 [109]. HPF also influences pro-inflammatory and immunological responses of microglia that are involved in the progression of neuropathological disorders. Actually, Kraus et al. reported that HPF significantly suppresses NF-κB activity and strongly inhibits iNOS expression in N11 and BV2 mouse microglia and RAW 264.7 macrophages treated with LPS [112]. Additionally, 1 µM HPF triggers differentiation in primary cultures of human keratinocytes and in derived HaCaT cell lines and inhibits their proliferation [122]. Pretreatment with SJW extract preserved PC12 cell line from H_2_O_2_-induced ROS generation and damage in a concentration-dependent manner (1–100 µg/mL) [123]. Feisst and Werz [124] found that HPF at very low concentration (IC_50_~0.3 µM) interferes with the induction of oxidative burst in polymorphonuclear leucocytes (PMN) by rather suppressing N-formyl-methionyl-leucil-phenylalanine (fMLP)-induced ROS production, than acting as a ROS scavenger. The authors reported that, after fMLP-elicited PMN stimulation, HPF is able to hinder ROS generation, by targeting an unknown component within the signaling cascade leading to Ca^2+^ mobilization [124].

Moreover, SJW and HPF exert potent anti-inflammatory effects in several animal model of acute and chronic inflammation by downregulating the expression or activity of inflammatory mediators and lowering ROS production. HPF attenuated microglial activation, p65 NFκB phosphorylation, and suppressed TNF-α expression in rat piriform cortex following status epilepticus [125]. HPF protected neuronal cells against aluminum maltolate-induced neurotoxicity by inhibiting ROS formation and enhancing superoxide dismutase and glutathione peroxidase activities [126]. In our laboratory, we have shown in various animal models, that SJW (30 mg/kg) prevents or attenuates tissue damage [127,128,129,130,131]. Acute lung inflammation induced by carrageenan is mediated by ROS production and activation of the redox-sensitive transcription factors NF-κB and STAT-3. SJW protect mice from induced pleurisy by reduction of PMNs infiltration in lung tissues and subsequent lipid peroxidation, by decrease of TNF-α and IL-1β production and nitro-tyrosine formation, as well as by inhibition of NF-κB and STAT-3 activity [127]. This finding is consistent with data reported in various other studies dealing with rodent models of carrageenan-induced paw edema, which was markedly reduced upon oral administration of SJW extract [132,133,134]. Zymosan administration in mice also resulted in excessive ROS formation by activated PMNs and lipid peroxidation in plasma, intestine, and lung [128]. In zymosan-injected mice pre-treated with SJW, we observed an increase in the intracellular amounts of both GSH and anti-oxidant enzymes associated with the reduction of iNOS expression as well as of ROS and nitro-tyrosine levels as compared to untreated controls [128]. Furthermore, SJW extract was able to mitigate cerulean-induced pancreatitis in mice by inhibiting edema, neutrophil infiltration and levels of intercellular adhesion molecule (ICAM)-1 and nitrosylated proteins in the injured pancreas, finally reducing the mortality of cerulean-treated animals [129]. In addition, SJW displayed a pronounced protective role against gastro-enteric inflammatory injury, as observed in rat gastric mucosa damaged by indomethacin administration [135] or in colonic mucosa after induction of experimental inflammatory bowel disease [136]. SJW was also able to protect rat intestinal epithelial architecture caused by irinotecan-elicited inflammation, likely dependent on the fact that SJW pretreatment limited the cytokine increase induced by irinotecan administration in the intestine and liver [137].

In summary, HPF-containing SJW extract or HPF can attenuate inflammatory response and subsequent tissue injury in several cell types and animal models by modulating a number of potentially harmful processes triggered by inflammatory signaling and ROS generation. 

### 5.2. Molecular Mechanisms of HPF Related to Its Protonophore Activity 

It has been suggested [96] that the molecular mechanism(s) underlying HPF effects toward various intracellular targets could be due to the fact that this phloroglucinol behaves as a protonophore, that is, as explained above, capable of increasing proton conductance of biological membranes. Interestingly, studies on the structure-activity relationship indicate that the protonophore activity of a compound, in most cases, is a linear function of the partition coefficient in the octanol and water (ow) system (Log K_ow_) and also depends on its acidic dissociation constant (pK_a_), an index of acidity strength [92]. For instance, the partition coefficient of several polyphenols ranges from 1 to 3 and their pK_a_ ranges from 4 to 9 [28]. Definitely, HPF is much more lipophilic than the majority of polyphenols as it displays a Log K_ow_ > 10, meanwhile its pK_a_ value of 6.3 allows effective buffering of pH changes in a neutral environment [138]. Both values represent ideal chemical features for a compound to act as a protonophore. Indeed, the ability of HPF to increase proton conductance of plasmatic and organelle membranes was firstly disclosed by Roz and Rehavi [139] to explain its inhibitory action on neurotransmitter reuptake by a non-competitive mechanism. Taking into account that the proton gradient, induced by V-ATPase, is the major driving force for vesicular monoamine uptake and storage, these authors showed that HPF (at 0.4–1 µM), alike the synthetic protonophore carbonyl cyanide 4-(trifluoromethoxy)phenyl-hydrazone (FCCP), is able to dissipate the pH gradient across the synaptic vesicle membrane [139]. Moreover, Froestl et al. [140] have linked the HPF protonophore activity to its aptitude to enhance amyloid precursor protein extrusion from the cells, process that is highly depending on intracellular pH. In this model, HPF, by affecting proton content, can indeed protect neurons against development of Alzheimer’s disease [140,141]. Sell et al. [96] confirmed that HPF, due to its physico-chemical properties, is easily incorporated into the membrane lipid bilayer, where it can function as a protonophore, triggering proton crossing regardless the involvement of any channel protein. Finally, in experiments carried out in isolated brain mitochondria, Tu et al. observed that HPF, likewise the protonophore and uncoupler FCCP, cause a concentration-dependent drop in the mitochondrial membrane potential [142]. Thus, HPF is also able to affect mitochondrial function, as well as mitochondrial (mt)ROS production [142].

## 6. Antitumor Activity of SJW Extract and Its Component HPF

### 6.1. Protective Effects of SJW and HPF against Carcinogenesis

The above described capability of SJW extract and HPF to defend normal cells and tissues from injurious stimuli could be the rationale for their preventive anti-tumor activity. As already mentioned, HPF suppresses 5-LO and COX-1 activity and PGE_2_ production both in human whole blood at low concentrations range (0.03–2 µM) and in a number of in vivo models [107,108,118,119]. Actually, inhibition of eicosanoid synthesis provides a molecular basis for not only the anti-inflammatory but also the anti-carcinogenic properties of HPF [119]. Indeed, excessive PGE_2_ formation has been found associated with tumorigenesis of colon, breast, prostate, and lung carcinoma [119]. Furthermore, overexpression of 5-LO protein was observed in cancer cell lines and tumor specimens [143]. 

Mainly due to their inhibitory action on inflammation-generated byproducts, SJW and HPF can also help preventing genotoxic effects. Actually, the anti-genotoxic ability of HPF has been verified by measuring the amount of bacterial gene mutations (Ames’ test), the occurrence of DNA strand breaks in human lymphocytes (comet assay) and the induction of chromosome aberrations in a mammalian cell line [144]. The results showed that HPF (at a concentration of about 1 µM) displays indeed anti-mutagenic activity as well as DNA protective effect against zeocin-induced single- and double-strand breaks and reduction of chromosome aberrations in human liver cells exposed to benzo[a]pyrene (B[a]P) or cisplatin. This suggests that HPF could de facto contribute to reduce human environmental risk [144]. The SJW- and HPF-dependent prevention of ROS generation, observed in different in vitro and in vivo inflammatory models [123,124,126,127,128,145,146], may account for their protective effect against genotoxic insults. In particular, the HPF ability to decrease ROS production could be crucial to prevent the genotoxic action of zeocin, that was reported to cause DNA damage in two breast cancer cell lines mainly by increasing intracellular ROS level [147,148]. Furthermore, the aforementioned protonophore action of HPF, by allowing protons to re-enter the cytosol and thus hinder intracellular alkalization, might explain the HPF protective effect on B[a]P-induced carcinogenesis, reported by Imreova et al. [144]. In fact, intracellular alkalization was detected in rat hepatic epithelial F258 cells quickly upon exposure to B[a]P, resulting in alteration of pH dynamics in different cell compartments including mitochondria and affecting mitochondrial function [149]. 

A protective action against oncogenesis has also been shown by using SJW total extract. In a mouse experimental model of colorectal carcinogenesis induced by azoxymethane, Manna et al. [150] reported the preventive potential of a dietary supplementation with SJW extract. Azoxymethane induces alkylation of DNA leading to mutations and tumorigenesis [150]. Diet enrichment with 5% SJW decreases the incidence of colorectal polyps and large tumors in azoxymethane-treated mice, finally improving overall animals’ survival. In this model, at molecular level, it has been observed that NF-κB and ERK1/2 pathways are hampered by SJW, with a reduction in the mRNA expression level of NF-κB-elicited genes, such as TNF-α, IL-1β, MMP-7 and -9 and iNOS [150]. These results clearly indicate that preventive administration of SJW can decrease both the occurrence of colorectal tumors and the associated induction of inflammatory signaling [150]. Interestingly, the same mouse model of colorectal carcinogenesis has recently been showed to depend on NOX/ROS activity, since a low dose of diphenyleneiodonium (DPI), a NOX inhibitor, prevents the formation of azoxymethane-induced adenomatous polyps and inhibits the intestinal inflammatory response [151]. Mechanistically, DPI decreases ROS production in the colon, resulting in inhibition of TNF-α, IL-6 and monocyte chemoattractant protein-1 (MCP-1) production, as well as ERK1/2, STAT-3 and NF-κB signaling, finally exerting a strong anti-inflammatory effect [151]. Thus, it is likely that DPI and SJW exert protective effects against azoxymethane-elicited colorectal carcinogenesis with a similar mechanism, i.e., by interfering with ROS-mediated inflammatory signaling. 

It is meaningful to remark that the presence of HPF in SJW extract can affect the pharmacokinetics of many co-administered drugs by inducing a number of liver cytochrome P450 (CYP) isozymes, such as CYP3A4, CYP2C19, CYP2D2 [152]. It has been shown indeed that HPF is a potent ligand for the pregnane X receptor (PXR), which is the principal transcriptional regulator of CYP3A enzyme expression [152]. Notably, several CYP isozymes are implicated in the detoxification of xenobiotics, or otherwise in the biotransformation of pro-carcinogens requiring metabolic activation to exert their genotoxic effects [153,154]. Nevertheless, at variance of most in vitro studies showing carcinogen activation by CYPs, in vivo experiments performed after gene deletion of P450 isozymes have often revealed that these metabolizing enzymes have a major role in detoxification rather than activation of carcinogens [154]. For instance, a recent study of Beyerle et al. [155] reported downregulation of xenobiotic-metabolizing enzymes, including CYP3A4, in human colorectal cancer tissues with respect to normal mucosa samples. Thus, the activation of CYP isozymes involved in carcinogen detoxification might be another mechanism by which HPF can function as a tumor preventive phytochemical agent. 

It has to be highlighted that the protective effects of SJW against colon carcinogenesis can occur also in humans. Remarkably, a very large epidemiologic study in USA (including 77,000 participants monitored for 5 years) explored whether the regular employment of various herbal supplements would lessen the risk for lung or colorectal cancers. The results showed that continuative use of SJW was associated with a 65% decrease in risk for colorectal cancer, although no protective effect was observed with regard to lung cancer [156].

It is worth mentioning that SJW could prevent tumor onset also by virtue of its antimicrobial and antiviral activities. For instance, quite low concentrations of an alcoholic SJW extract have been shown to exert powerful protection against *Helicobacter pylori*, a pathogen that is known to increase the risk of some forms of stomach cancer [157]. In a more recent study, the antimicrobial activity of SJW against *H. pylori* was confirmed by the observation that low concentrations of SJW extract were able to rapidly kill a high percentage of most strains of this bacterium [158]. SJW is also able to inhibit the growth of viruses implicated in the development of some cancers, such as hepatitis B virus (HBV) whose long-term infection could indeed result in development of cirrhosis and hepatocellular carcinoma. SJW ethanol extract has been reported to have strong inhibitory effects on HBV in vitro by decreasing the expression of HBV DNA and the secretion of HBV antigens [159]. 

### 6.2. Effects of SJW and HPF on Cell Proliferation and Apoptosis 

When tumor has already grown, SJW and HPF can still exert therapeutic effects by down-modulating survival signaling and/or inducing apoptosis. Remarkably, in rats injected subcutaneously with MT-450 breast cancer cells and administered HPF daily at the site of the cell transfer, in vivo tumor growth was inhibited in the absence of any side effect [160].

Ten human and six rat cancer cell lines, derived from melanoma, breast and ovary carcinoma, prostate and pancreas tumors, glioblastoma, sarcoma and T cell leukemia, were found to be very sensitive to the antitumor effects of hyperforin-DCHA (in the range 5–20 µM) [160]. HPF-DCHA slowed down tumor cell proliferation and, at the highest concentration used in this study (20 µM), induced apoptosis as well as caspase-9 and -3 activities [160]. Apoptosis was triggered by a loss of mitochondrial transmembrane potential (Δψ_m_) very early after HPF exposure, accompanied by a rapid release of cytochrome (Cyt) c, as assessed in a mitochondria-enriched cell fraction [160]. Recently, Hsu et al. [161] reported that in two glioblastoma cell lines, HPF prompts apoptosis, increase of cytosolic [Ca^2+^], loss of Δψ_m_, suppression of EGFR/ERK/NF-κB signaling, and decrease of anti-apoptotic proteins expression. Wiechmann et al. [162] also showed that HPF can directly impair viability of mitochondria isolated from HL-60 cells by affecting mitochondrial proton-motive force. Again, in hepatocellular carcinoma cell line, HPF significantly inhibits cell viability and cyclin D1 expression and induces loss of Δψ_m_ and downregulation of the anti-apoptotic proteins fetal liver LKB1 interacting protein c (c-FLIP), X linked inhibitor of apoptosis protein (XIAP) and myeloid leukemia cell differentiation protein (Mcl-1), finally triggering apoptosis [163]. 

By acting as a protonophore in the plasma membrane, HPF can counteract cytosolic alkalization, thus allowing apoptosis. Nevertheless, for the achievement of the apoptotic goal, the major target of HPF appears to be the inner mitochondrial membrane, whose hyperpolarization in cancer cells protects them from apoptosis [28]. In fact, HPF-induced severe dissipation of Δψ_m_ provokes pore formation and Cyt c release, sensitizing cancer cells to death [28]. Moreover, the fall in mitochondrial membrane potential and the consequent lowering of mtROS hinders both mitogen signals triggered by growth factors and activation of survival kinases, including ERK1/2 and Akt [48]. As reported above, mitochondrial membranes incorporate lipophilic HPF [96,164], so that, while in normal condition the inner membrane is impervious to protons, in the presence of HPF, protons can shuttle into the matrix following their concentration and electric gradients (as described in Figure 2), with consequent breakdown of Δψ_m_. Other natural active compounds, such as curcumin, epigallocatechin gallate, honokiol, myricetin, urolithin A, moronone, nemorosone, may also exert, at least partially, antitumor action by dissipation of Δψ_m_ [28,95,165], as do the synthetic uncoupling molecules [166]. In this regard, the protonophore carbonyl cyanide m-chlorophenyl hydrazone (CCCP) directly interferes with mitochondrial function and induces apoptosis [167,168]. It has also been reported that uncoupling agents such as 2,4-dinitro-phenol and others, acting as protonophores, can prevent inflammatory response even at low concentrations, by inhibiting TNF-α-dependent activation of NF-κB signaling [93,169]. Hence, NF-κB is confirmed to be a crucial target of SJW and HPF antitumor action also through mechanisms involving an uncoupling/protonophore activity that HPF shares with classical uncouplers. Actually, it has been documented that SJW extract promotes apoptosis and decreases NF-κB protein level in MCF-7 cells [170] and that SJW oil inhibits NF-κB activation in human chronic myelogenous leukemia K562 cells [171]. HPF decreases the expression level of anti-apoptotic proteins and induces apoptotic cell death through blockade of NF-κB activity in non-small cell lung cancer [147], bladder cancer [172] and U-87 and GBM-8401 glioblastoma [161] cell lines. In order to clarify the molecular mechanism leading to NF-κB inhibition by HPF, it should be reminded that this transcription factor can be activated by inflammatory mediators through ROS as second messengers [57,59]. Thus, HPF, by blocking NOX/ROS-dependent signal transduction elicited by cytokines and growth factors, and/or mtROS production by decreasing Δψ_m_, could affect NF-κB activation and its downstream signaling pathway. 

Many pro-apoptotic proteins are encoded by NF-κB-responsive genes. Notably, several investigations document a pro-apoptotic effect of SJW and HPF depending on a complex modulation of pro- and anti-apoptotic members of the B cell lymphoma 2 (Bcl2) family of proteins. For instance, in MCF-7 human breast cancer cells, SJW extract induces apoptosis by increasing the pro-apoptotic proteins Bcl 2 associated X (Bax) and Bcl 2 antagonist of cell death (Bad) and decreasing the anti-apoptotic proteins Bcl-2, B cell lymphoma extra-large (Bcl-xL), and phosphorylated form of Bad (pBad), finally leading to caspase 7 activation and cell death [173]. In human myeloid tumor cells, Merhi et al. [174] show that HPF inhibits Akt kinase activity, determining dephosphorylation of Bad, an Akt substrate, and activation of its pro-apoptotic function. The authors conclude that HPF, as a negative regulator of Akt, could represent an interesting novel approach for treatment of AML and other malignancies. In human K562 T cell leukemia and U937 lymphoma cell lines, Hostanska et al. confirm the anti-carcinogenic property of HPF that triggers a caspase-dependent apoptotic cell death [175]. The HPF antitumor activity has been also tested in primary cells of hematological malignancies, i.e., in leukemic cells from B-cell chronic lymphocytic leukemia (B-CLL) patients [176]. HPF induces apoptosis in the B-CLL cells by eliciting a drop in mitochondrial transmembrane potential and activation of caspase 3. In this study, the anti-apoptotic factors Bcl-2 and Mcl-1 as well as the protein expressions of iNOS and p27^kip1b^ are downregulated [176]. In the B-CCL cells, Zaher et al. [177] show a strong correlation between the upregulation of Noxa protein expression and HPF-elicited cell apoptosis, that is in fact partially inhibited by RNA-interfering Noxa silencing. Notably, the pro-apoptotic activator Noxa, by binding to Mcl-1 and neutralizing its anti-apoptotic action, is involved in B-CCL cells death [178]. HPF is also able to elicit growth arrest and caspase-dependent apoptosis in acute myeloid leukemia (AML) cell lines and in primary AML cells, in which the apoptotic mechanisms are again traced back to mitochondrial membrane potential loss, Noxa overexpression and Mcl-1 downregulation [174]. 

As the ability of HPF to regulate the balance between pro- and anti-apoptotic proteins has been so frequently associated with mitochondrial membrane potential loss, due to its protonophore activity, it is actually surprising that in many experimental models this mechanism has not yet been extensively investigated. However, all studies which measured both apoptosis activity and mitochondrial membrane potential found a negative correlation between these two phenomena [160,162,163,174,176], suggesting a close link between HPF mitochondrial protonophore action and programmed cell death susceptibility in cancer cells. 

**Figure 3:**
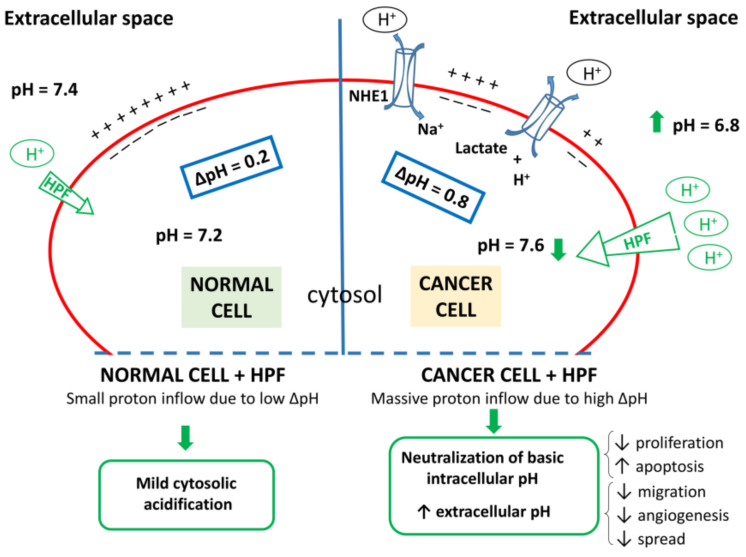
Proton dynamics in normal and cancer cells. Left: normal cells display mild difference in proton concentration between extracellular space and cytosol (ΔpH~0.2). Right: Differently from normal cells, cancer cells show high activity of a number of channels that regulate pH (NHE; Hv; V-H^+^/ATPase; Na^+^/HCO_3_^−^ cotransporter; H^+^/K^+^-ATPase and H^+^/lactate cotransporter, two of them being represented as examples), which results in increase of proton concentration in the extracellular space combined with cytosol alkalization (ΔpH~0.8). In the presence of HPF, due to the protonophore activity of this compound, proton conductance increases. In both cell types exposed to HPF, the negative charges of the cytosolic side of plasma membrane foster a proton influx, which is small in normal cells and large in cancer cells displaying higher ΔpH. Thus, cancer cells exposed to HPF can import a larger amount of protons which counteracts intracellular alkalization and extracellular acidification, both hallmarks of malignancy. Green arrows and outlines refer to the putative effects of HPF.

It is worth to highlight that the HPF pro-apoptotic action is selectively observed in cancer cells, whereas healthy cells are much less sensitive to HPF cytotoxicity. It is known indeed that the same concentration of HPF that induces clear-cut damage in B-CLL cells is totally unable to compromise viability of human B lymphocytes from healthy donors [176]. The reason why normal cells are more resistant to HPF cytotoxic action could be related to their intrinsic differences in metabolism, pH and ionic concentrations in the cytosol and organelle compartments with respect to malignant cells. For instance, in normal cells the negative cytosolic side of the plasma membrane facilitates proton influx, although this influx is attenuated by the mild pH gradient in the opposite direction, resulting in only a faint proton entry in the presence of a protonophore such as HPF (Figure 3). Instead, in malignancy, the negative plasma membrane charges together with a reversed pH gradient, due to the highest activity of proton channels that extrude proton from the cells, determine a substantial driving force for robust proton influx. Thereby, when the proton conductance is empowered by the presence of a protonophore agent, a significant and persistent cytosolic proton influx occurs in cancer cells. Alike other protonophore, HPF elicits proton influx in tumor cells restoring cytosolic acidity and allowing apoptosis. At the same time, extracellular tumor microenvironment would be impoverished in proton concentration (i.e., pH_e_ tends to neutrality), thus impeding extracellular matrix digestion, tumor cells migration and invasiveness (Figure 3).

This hypothesis must be experimentally verified in the case of malignant cells, but is consistent with the findings of Sell et al. [96] in both plasmatic membrane of normal cells (primary microglia and chromaffin cells) and synthetic lipid bilayer (devoid of any channel protein), in which HPF was shown to mediate a proton conductance due to its intrinsic protonophore activity and resulting in proton entry into the cell and cytosol acidification. Notably, the extent of proton influx, in the presence of HPF, is directly depending on the proton gradient between the two sides of the membrane [96]. These authors also demonstrated, by measurement of capacitance changes in the presence of various HPF concentrations, that this lipophilic compound accumulates in the membrane, which might explain the effects of even low doses of HPF [96] and the persistence of the protonophore activity also when SJW or HPF is no more available in the cell culture medium [113]. 

At the present state of our knowledge, the hypothesis that HPF can change the proton flux in function of the proton gradient could explain why HPF is not cytotoxic for normal cells, whereas it can induce apoptosis in cancer cells. In cancer cells, both at the plasma membrane level, in which ΔpH favors high proton influx, and at the hyperpolarized inner mitochondria membrane, the presence of HPF could counteract malignant phenotype, or at higher concentration, promote cell death by collapsing inner mitochondrial membrane.

In general, other effects of natural products could be related to their uncoupling activity. As an example, curcumin, that can act as an uncoupler, is able to increase AMP/ATP ratio, thereby activating AMP-activated protein kinase (AMPK) [179]. In this way, curcumin can inhibit mTOR that decreases Ser727 phosphorylation of STAT-3, blocking cell proliferation [180]. On the other hand, activated AMPK stabilizes the tumor suppressor p53 favoring cellular sensitization to mitochondrial apoptosis [181]. Similarly, in HL60 leukemic cells, HPF and myrtucommulone, another phloroglucinol derivative, directly interact with the mitochondrial membrane, decreasing Δψ_m_, lowering the ATP level and activating AMPK [162]. Moreover, SJW treatment, in the concentration range of 10–50 µg/mL, enhances pAMPK and decreases pAkt, suppressing growth of breast cancer MCF7 cells. SJW is also able to repress protein synthesis by decreasing the phosphorylation levels of both mTOR and its downstream substrate 4E-BP1 [173]. 

### 6.3. SJW and HPF Affect Neo-Angiogenesis, Tumor Cell Invasion and Metastasis.

The capability of SJW and in particularly HPF to inhibit angiogenesis, cell invasion and metastasis has been largely investigated. 

Martinez-Poveda et al. [182,183] showed that HPF can inhibit angiogenesis both in bovine aortic endothelial (BAE) cells in vitro and in chorioallantoid membrane in vivo. HPF also inhibits MMP-2 and urokinase secretion from BAE cells and restrains their invasive capability in a Matrigel layer. The authors claim that HPF could be considered a promising anti-angiogenic natural product interfering with key events in angiogenesis at concentrations (10 µM) that do not cause endothelial cell death [183]. The anti-angiogenic activity of HPF has been confirmed by many other studies. Lorusso et al. [184], using hyperforin-DCHA in human umbilical vascular endothelial cells (HUVEC), showed that HPF induces cytostatic but not cytotoxic effects, significantly reducing HUVEC cell migration triggered by chemoattractant stimuli. Again, the authors showed that in the presence of 1–3 µM HPF, the chemokine-elicited migration of neutrophils and monocytes as well as the TNF-α-stimulated nuclear translocation of NF-κB were markedly inhibited in HUVEC cells [184]. In an in vivo mouse model of conspicuous angiogenesis induced by subcutaneous injection of Matrigel implants, HPF potently prevents neo-vascularization as well as expression of pro-angiogenic IL-8 and MCP-1 chemokines [184]. In addition, the growth of Kaposi’s sarcoma (a highly angiogenic tumor) in HPF-treated mice is markedly diminished both in size and vascularization with respect to untreated controls. The authors conclude that HPF can block many crucial events in tumor angiogenesis, most likely by inhibition of inflammatory signaling, thus opening new perspectives in the treatment of solid cancers [184]. 

Likewise, Rothley et al. [185] reported that HPF and its more stable analog aristoforin can block cell cycle and proliferation of either arterial or lymphatic endothelial cells at 5 µM concentration, whereas they induce apoptosis at higher concentrations (>10 µM). The authors analyzed the effect of these compounds in a rat model of tumor-induced lympho-angiogenesis, obtained by subcutaneous injection of MT-450 malignant cells. When HPF or aristoforin is administered daily in the peri-tumor area for two weeks, the lymphatic capillary outgrowth is significantly reduced, suggesting that these bio-active compounds can limit tumor-induced lympho-angiogenesis also in vivo [185].

Angiogenesis involves a tight regulation of multiple signaling pathways, among which VEGF is the most prominent effector, although PDGF and bFGF also play a role [186]. It is well-known that ROS signaling is linked to angiogenesis and involves induction of several kinase pathways as MAPKs, PI3K, Akt, and activation of transcription factors, including HIF-1, NF-κB, STAT3 [187]. For instance, ROS determine HIF1 stabilization, which in turn increases the transcription of many angiogenic genes, including VEGF [187]. Additionally, the promoters of the pro-angiogenic chemokines, such as IL-8 and MCP-1, contain binding sites for NF-κB which is regulated in a redox dependent manner [188]. Notably, NOX-derived ROS are required for the angiogenic response induced by various growth factors, with also the contribution of mtROS. It must be recalled that extracellular acidification supports angiogenesis, since it regulates VEGF expression in a both transcriptional and post-transcriptional manner, thus suggesting that an acidic tumor microenvironment can contributes to cancer progression [189]. Moreover, the G protein-coupled receptor (GPR)-4 expressed on endothelial membrane acts as a sensor of extracellular protons and stimulates intracellular signaling. Indeed, GPR4-deficient mice show strongly reduced responses to VEGF-driven angiogenesis, with a reduction of tumor growth that is correlated with impaired vessel structure and lower VEGF receptor 2 level [190]. Therefore, it can be argued that a change in proton concentration driven by the protonophore activity of HPF could affect angiogenic signals.

Interestingly, the contribution of enzymes such as metalloproteinase to tumor invasiveness and metastasis can be potentiated by their proton concentration dependency. Actually, the expression and secretion of MMP-9 increases at lower pH and higher pHi [84]. Additionally, acid-activated proteases, such as cathepsin B, cleave latent MMPs into active enzymes. These examples of pH-dependent proteins reveal potential therapeutic targets and strategies based on using changes in pH to control tumor metastatic process [84], as recently confirmed by Robey et al. [191], who were able to inhibit experimental and spontaneous metastases by increasing systemic buffering capacity and tumor pHe through oral bicarbonate administration to mice.

Anyway, HPF, besides showing anti-angiogenic activity, inhibits the MMP-9 production in B-CLL [192] and prevents the formation of microtubules in bone marrow endothelial cells, so that its therapeutic use in B-CLL patients has been suggested [192]. Furthermore, non-cytotoxic HPF concentrations can hinder cell invasiveness by downregulating the activities of MMP-2 and -9, elastase and cathepsin G, highly expressed in inflammatory and tumor cells [116]. Such downregulation is most likely dependent on HPF-induced inhibition of the constitutively activated ERK1/2 in malignant cells, responsible for the enhancement of MMPs expression [193]. In this way, HPF can counteract the capability of tumor cells to digest components of extracellular matrix, thereby impairing their spreading. Indeed, HPF has been shown to significantly reduce the number of lung metastatic foci in mice receiving colon carcinoma or melanoma cells, suggesting that this natural compound can effectively prevent cancer growth and metastatic spread in vivo [116].

Finally, it should be mentioned that an observational study suggests that SJW can exert anti-tumor effects also in humans. A clinical report described the beneficial effect of SJW in two cases of colon cancer and one of duodenal adenocarcinoma. None of the cases received neo-adjuvant chemo-radiotherapy or additional treatment, but only SJW oil (one teaspoon in the morning) before surgery. In each patient, an intense lympho-plasmocytic reaction occurred, resulting in a fibrosis surrounding the tumor, that created a defensive shield against the penetration of the tumor cells into underlying tissue [194]

## 7. Conclusions

HPF, the main active component of SJW, that has been shown to be the major responsible for the antidepressant effect of this plant extract, is provided of additional advantageous properties, including anti-inflammatory, antiangiogenic and antitumor activities.

**Figure 4:**
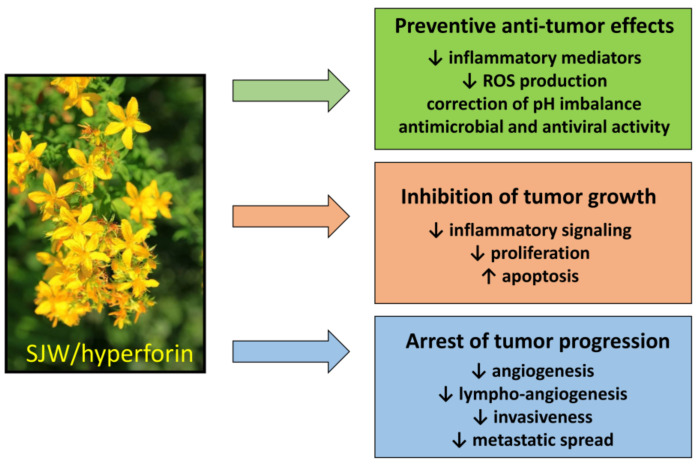
Antitumor effects of SJW and HPF. SJW and HPF display many antitumor effects with regard to both prevention and therapy of neoplastic diseases by acting on many different intracellular targets.

The beneficial effects of HPF-containing SJW extract against cancer are summarized as follows (Figure 4).

1. Tumor prevention. Regular use of SJW reduces cancer risk, by preventing the genotoxic effect of carcinogens [144]. This protective action is essentially based on the ability of HPF to slowdown inflammatory mediators and regulate ROS production and/or pH imbalance, resulting in counteraction of malignant phenotype. It should be emphasized that preventive dietary supplementation of SJW has been found to reduce the risk of colorectal cancer also in humans [156].
2. Effects on tumor growth and spread. The anti-inflammatory and anti-angiogenic effects of SJW/HPF, presumably due to inhibition of cytokine and chemokine production and hindrance of their downstream signaling, can protect from tumor expansion. Additionally, the protonophore property of HPF can avoid the acidification of the tumor extracellular milieu, impairing neo-angiogenesis and metalloproteinases activity and thereby tumor invasiveness and metastatic spread [116]. The HPF ability to drop off mitochondrial membrane hyperpolarization and consequently mtROS generation inhibits cell proliferation and favors apoptosis induction. Indeed, the pro-apoptotic effect of SJW/HPF is well documented in many malignant cell lines or in animal tumor models and appears determined by the imbalance between pro- and anti-apoptotic protein expression, even though the concentrations of HPF capable of inducing apoptosis by such mechanism are quite high (at least 5–10 µM) and difficult to be achieved in clinical treatment. Nevertheless, it is well known that proliferating cancer cells display higher Δψ_m_ than normal cells, and that positive correlation exists between the malignancy grade of cell clones and their mitochondrial potential [195]. Consequently, malignant cells are more sensitive than normal cells to even small changes of the mitochondrial electro-chemical gradient. Thus, low doses of SJW/HPF, likely insufficient to induce cancer cell death, can nevertheless block ROS-elicited tumor growth and spread by hindering activity of pro-survival protein kinases and angiogenesis.
3. Bioavailability and broad spectrum of action. Furthermore, SJW/HPF, for their large bioavailability, persistence of protective benefits and substantial absence of adverse effects, are natural products of biological relevance for tumor prevention and treatment. As cancer therapy requires a multifactorial strategy, SJW/HPF treatment can actually meet this requirement, due to their pleiotropic effects against many different molecular targets along signaling pathways crucial for tumor growth and progression. Additionally, SJW/HPF, acting against different types of tumor cells, can be a broad-spectrum anti-tumor compound and should be tested in association with current chemotherapy drugs to achieve additive effects.
4. Limitations. The therapeutic use of HPF-containing SJW extract as anti-depressant has confirmed its very good tolerability and the paucity of adverse effects. However, a major concern of SJW/HPF treatment regards the possible occurrence of drug-drug interaction, due to HPF high affinity binding to PXR, resulting in increased expression levels of cytochrome P450 isoenzymes [141]. Actually, by activating CYP3A4, HPF can enhance drug metabolism and excretion, thereby reducing the effectiveness of a number of chemotherapeutic agents. Thus, the association of SJW/HPF to a chemotherapeutic drug should be thoughtfully decided and monitored to ensure that the drug efficacy is maintained. Very interestingly, several phloroglucinol derivatives devoid of PXR binding activity, have recently been characterized [196]. These HPF derivatives have already been proven to maintain antidepressant activity, although antitumor properties have not yet been tested and should be investigated in a near future.

On the basis of the studies presented in this review, SJW extract and its component hyperforin, already safely employed in antidepressant therapy, hold considerable promise to exert relevant beneficial effects as anti-tumor phytochemicals. These natural compounds have a remarkable potential for both prophylactic and therapeutic use against tumor development by mechanisms that involve modulation of ROS production and action along different steps of carcinogenesis as well as regulation of proton dynamics in cancer cells. Although further studies are undoubtedly required for a full clarification of mechanisms and effects, ample experimental evidence in vitro and in vivo already point out that SJW and HPF can play a remarkable role as nutraceutical supplement or in association with chemotherapy in the challenging fight against cancer.
